# Why is endothelial resilience key to maintain cardiac health?

**DOI:** 10.1007/s00395-022-00941-8

**Published:** 2022-07-14

**Authors:** Lukas S. Tombor, Stefanie Dimmeler

**Affiliations:** 1 Institute of Cardiovascular Regeneration, Goethe University Frankfurt, Frankfurt, Germany; 2 Faculty for Biological Sciences, Goethe University Frankfurt, Frankfurt, Germany

**Keywords:** Endothelial, Plasticity, Endothelial-to-mesenchymal transition, Myocardial infarction, Resilience, Cardiac remodeling, Microenvironment

## Abstract

Myocardial injury as induced by myocardial infarction results in tissue ischemia, which critically incepts cardiomyocyte death. Endothelial cells play a crucial role in restoring oxygen and nutrient supply to the heart. Latest advances in single-cell multi-omics, together with genetic lineage tracing, reveal a transcriptional and phenotypical adaptation to the injured microenvironment, which includes alterations in metabolic, mesenchymal, hematopoietic and pro-inflammatory signatures. The extent of transition in mesenchymal or hematopoietic cell lineages is still debated, but it is clear that several of the adaptive phenotypical changes are transient and endothelial cells revert back to a naïve cell state after resolution of injury responses. This resilience of endothelial cells to acute stress responses is important for preventing chronic dysfunction. Here, we summarize how endothelial cells adjust to injury and how this dynamic response contributes to repair and regeneration. We will highlight intrinsic and microenvironmental factors that contribute to endothelial cell resilience and may be targetable to maintain a functionally active, healthy microcirculation.

## Introduction

**Fig. 1:**
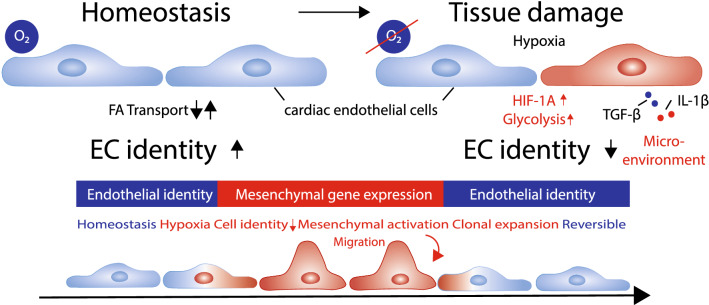
Endothelial cells identity changes after myocardial infarction. Cardiac microvascular endothelial cells maintain a tissue dependent cell identity program at baseline level. Tissue damage leading to hypoxic signaling and increase of glycolytic metabolic pathways together with increased concentrations of pro-inflammatory cytokines lead to loss of cell identity and expression of a mesenchymal gene program. While this induction favors migratory and proliferative responses in endothelial cells after infarction, mesenchymal activation is a temporary and reversible mechanism

In the adult heart, a tight interplay between multiple cell types ensures tissue integrity throughout lifetime. Fibroblasts are known to orchestra cardiomyocyte function, by providing structural scaffold and electromechanical support [25, 33]. Resident cells of the immune system degrade apoptotic waste and act as sentinel cells to detect small changes in the microenvironment [26, 61]. Proper contraction of the heart requires oxygen and nutrient supply, which is guided by the vasculature and its direct cellular interface to the blood, the endothelium. All these cellular functions are coupled to distinct molecular maintenance programs, which tightly control cell identity. This includes intrinsic mechanisms, which prime the transcriptional landscape of the cells, as well as extrinsic signals by surrounding microenvironmental factors [5, 6, 29]. Tissue damage as induced by myocardial infarction perturbs this steady state and enables individual cells of the heart exiting the maintenance and identity program. While these adaptive responses associated with increase in cellular plasticity are essential for tissue repair, the ability of cells to revert to the homeostatic phenotype is the key for long-term cardiac health (Fig. 1). This ability to return to the original state after a stress response is often referred as *cellular resilience*.

**Fig. 2:**
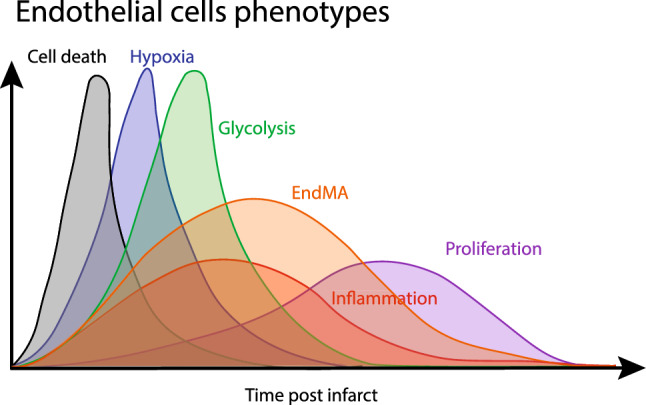
Timeline of endothelial phenotypes after myocardial infarction. Endothelial cells transiently respond and adapt to changes in the cardiac microenvironment. After the first wave of cell death, which occurs in obstructive and infarcted areas, the partial O_2_ decreases, leading to the onset of hypoxia. Later, cells induce glycolytic genes, which favors plastic phenotypes such as endothelial-to-mesenchymal activation (EndMA). Recently, the emergence of this mesenchymal phenotype has been associated with clonal expansion and proliferation. Endothelial activation and inflammatory signaling can be seen in multiple phases after myocardial infarction, as the endothelium interacts and responds to innate and adaptive immune reactions

**Fig. 3:**
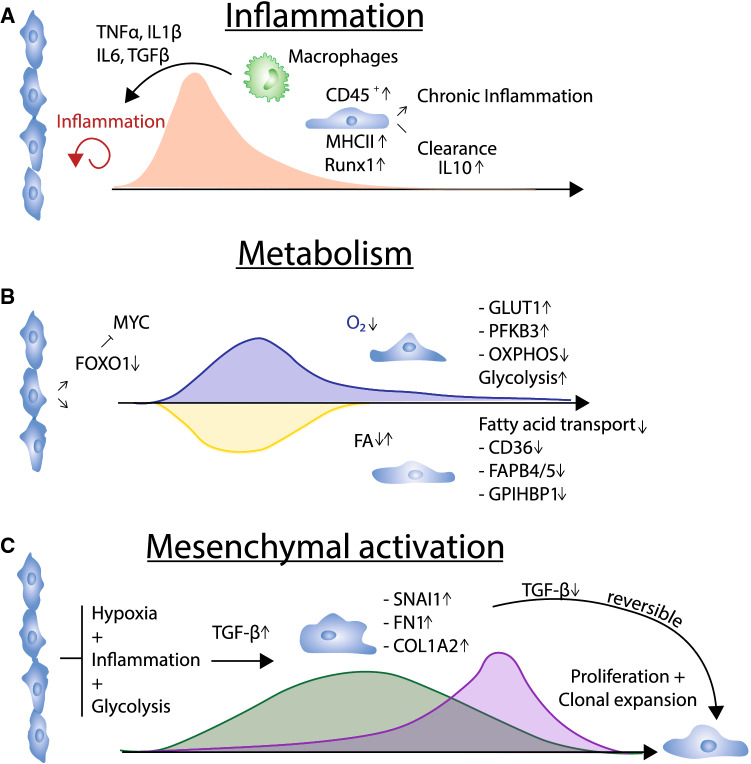
Transient phenotypes after myocardial infarction. **A** The emergance of innate immune reactions favors an inflammatory-induced phenotype which has been described to occur based on increased pro-inflammatory signaling (TNFα, IL-1β, IL-6). Also levels of TGF-β which can origin from multiple sources are associated with this phenotype. Endothelial cells show increased expression of hematopoietic marker CD45 and major histocompatibility complex (MHC) class II molecules. Whether this phenotype is cleared or leads to a chronic inflamed endothelium is debated. **B** Endothelial metabolic changes after myocardial infarction. Repression of FOXO1 leads to induction of MYC, which initiates a switch from fatty acid transport to glycolytic signaling. This induction is transient and reverts when normoxic conditions are restored. **C** Mesenchymal activation induces proliferation and clonal expansion. Hypoxia, inflammation and a glycolytic metabolism with increased TGF-β levels induce mesenchymal marker genes, such as *Snai1*, *Fn1* or *Col1a2.* Mesenchymal activation has been shown to induce clonal expansion in endothelial cells, leading to neovascularization. Upon loss stimulating TGF-β levels, endothelial cells revert back to a naïve phenotype.

**Fig. 4:**
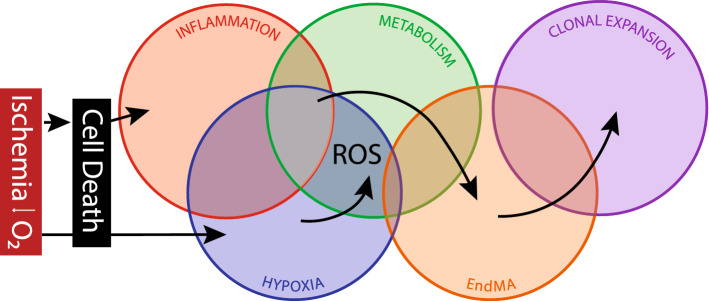
Overlapping combinatory signals lead to clonal expansion. The emergence of ischemia leads to a combinatorial reaction of events that favor clonal expansion and neovascularization. Cell death leads to the onset of inflammation which alters endothelial cells phenotype. In combination with hypoxic signaling and altered metabolism, cells can transiently activate a mesenchymal program, which leads to clonal expansion

The era of single-cell multi-omics significantly broadens our understanding of how dynamical stress responses change chromatin and transcriptional landscapes and induced a reconsideration of how we define cellular heterogeneity. Interestingly, studies of cardiovascular tissues rarely identified entirely new cell populations but highlight the presence of multiple states of cellular identities within the heart upon injury [6, 7, 26, 49, 65]. In contrast to previous reports on specialized progenitor pools, increasing evidence suggests that the tissue-resident microvascular endothelial cells (ECs) have a high plasticity and regeneration capacity. Transient changes of the transcriptome after injury contribute to the onset of neovascularization by clonal expansion [78], modulation of the inflammatory response [120], para- and autocrine signaling [116] or metabolic adaptations [62] (Figs. 2, 3, 4).

The ability of ECs to transiently respond, adapt but also recover maintenance functions strongly impacts long-term cardiac health. Its failure leads to endothelial dysfunction which impairs heart functions [146]. Here, we are summarizing why endothelial resilience is the key for cardiovascular repair and we review novel insights into EC plasticity.

## Endothelial plasticity after myocardial infarction

Myocardial infarction (MI) results in impaired tissue functionality, which in the long term results in chronic heart failure. Mechanistically, incomplete regeneration of infarcted areas leads to accumulation of fibrotic tissue and microvascular dysfunction. While most patients survive the acute hospitalization [59, 129], 20–30% of these patients develop heart failure within one year post MI [44, 114]. Here, successful reperfusion therapy within the first 90 min is decisive to prevent long-term loss of cardiomyocytes [45]. However, microvascular EC function can substantially suffer, resulting in reduced barrier function, permeability and inflammation [108], ultimately leading to endothelial dysfunction and impaired vascularization. Especially in early phases, ECs have remarkable plasticity and actively contribute to neovascularization and tissue remodeling. Here we are highlighting important adaptive responses of EC, including changes in phenotypes, after myocardial infarct. Of note, major insights derive from mice models in which short-time ischemia/reperfusion (I/R) injury or permanent ligation of left anterior descending coronary artery is used [128]. Both have limitations and may not fully recapitulate human pathophysiology. While I/R procedures are potentially mimicking the situation in patients more precisely, the experimental occlusion time largely determines the outcome, making studies difficult to compare. Permanent ligation models are more broadly used and provided many mechanistic insights into the naturally occurring pathophysiology, which leads to large infarcts and scar tissue. However, micro-embolization based on spontaneous erosion on plaque and obstruction of the capillary network as seen in patients with acute coronary syndrome is difficult to model in rodents [55]. Capillary obstructions lead to no-reflow phenomena and may induce distinct EC responses [39, 41]. Future analysis of human cardiac tissue from patients suffering myocardial infarction by single-cell technologies may help to validate the findings in mice, but will also shed light on how different clinical interventions or infarct size impact endothelial resilience.

### Endothelial responses to cell death, hypoxia and inflammation favor interactions with the immune system

Restriction of oxygen and nutrient supply after myocardial infarction represents a challenge for all cells in the damaged tissue, leading either to cell death or induction of survival genes. Cell death is induced in cardiomyocytes and interstitial cells within 30 min post infarction. Cells close to the endocardium are more prone to injury, leading to propagation of cell death toward outer regions at the epicardium [103]. Moreover, I/R with fluctuations in oxygen levels lead to accumulation of ROS. Notably, the levels of ROS in ECs appears to determine whether survival or apoptosis genes are activated [110].

Loss of oxygen not only induces cell death, but activates and stabilizes the transcription factor HIF-1A, which induces survival genes and controls metabolic adaptation. Induction of HIF-1A augments glycolytic metabolism and leads to higher expression of angiogenic factors, such as ANGPT1 and ANGPT2 or VEGF [67, 126]. This initiates neovascularization, which will be discussed in “Angiogenesis and proliferation”.

Inflammation starts when cells of the innate immune system recognize danger-associated patterns, which lead to activation of tissue-resident macrophages and neutrophils and production of pro-inflammatory cytokines and chemokines, such as TGF-β, IL-1β, IL-6, TNF-α. Thereby, damage-induced matrix degradation, production of reactive oxygen species (ROS) and complement activation are the three major drivers in the initiation of immune responses [62, 63]. Interestingly, onset of inflammation largely depends on activation of Toll-like receptor 2 and 4 in the heart [9, 94].

The endothelium responds to the pro-inflammatory environment by supporting immune cell infiltration specifically via degranulation of Weibel–Palade bodies containing P-Selectin [90] and by induced expression of ICAM1 and VCAM1 [26] which promotes leukocyte adherence. ECs additionally produce cytokines and chemokines within 24 h after infarction [124], which fuels the inflammatory activation. TNF-α as well as other paracrine or soluble factors, induce endothelial activation, apoptosis and ROS production, whereas NO biosynthesis is reduced, overall leading to a destabilization of the endothelial monolayer [54, 56, 57]. Moreover, endothelial activation promotes the local adhesion of thrombocytes by von Willebrandt factor [12, 130]. Pro-inflammatory activation and increased thrombocyte adhesion were also shown to occur in remote vessels, such as larger arteries after myocardial infarct. These remote vessel alterations were attributed to systemic activation of the endothelium, leading to higher risk for atherosclerotic after infarction [85].

Recently, ECs were discussed to facilitate immune modulation beyond these classical functions in innate immunity. Evidence of a plastic conversion toward “immune cell-like” phenotype (EndICLT) has been shown on single-cell level when comparing static and disturbed flow conditions in a mouse carotid ligation model [6]. This proatherogenic phenotype was associated with increased expression of typical markers of macrophages, including major histocompatibility complex II (MHCII). Specialized endothelium, like sinusoidal ECs in the liver, has antigen-presenting functions, and can act as sentinel cells, sensing bacterial infections and cross-prime T cells to activation [15, 112, 121]. In the heart, the presence of MHCII-positive ECs with antigen-presenting activities has been described already in the 1990s [106]. In addition, mitral valve ECs were shown to express the hematopoietic marker CD45 post infarction [11], which suggests that these EC may undergo a hematopoietic transition.

The endothelial and hematopoietic fate are tightly linked. In evolution, the emergence of an EC monolayer is hypothesized to occur from specialized circulating blood cells, so called amoebocytes, which epithelized the basal membrane and form a primitive blood tissue barrier [87]. Higher vertebrate ECs share a common progenitor with cells from the hematopoietic system, the hemangioblast, which likewise gives rise to endothelial and hematopoietic progenitors [43, 145]. This explains not only why endothelial-specific signatures, such as PECAM1, CD34 or VEGFR2 [28, 87], are still also found in cells of the hematopoietic system, but also might be the reason that pro-inflammatory cytokines, which have pleiotropic functions on immune cell activity also influence endothelial plasticity. Notably, the transcription factor RUNX1, known to maintain expression of identity genes in hematopoietic stem cells, has been shown to be upregulated in ECs after infarction [82]. While its role in angiogenesis and tissue repair remains controversial, there is evidence that RUNX1 is not only crucial in the formation of hemogenic ECs in development but also has functions in adult heart ECs [19]. Finally, emerging concepts suggest that ECs may fully convert into an immune phenotype after prolonged activation in chronic inflammation [63]. These findings may imply that cardiac ECs are privileged to share functions with hematopoietic cells, allowing to combine forces with immune cells in events of early acute damage but also on the long-run.

EC may not only partially transition to a hematopoietic fate, but myeloid cells may acquire endothelial cell features. Here, the presence of circulating endothelial cells progenitors (cEPCs) has been debated for long time and hint to interesting links between the endothelial and hematopoietic system [123, 127]. Original research dated back to the 1990s and early 2000s showed the existence of bone marrow derived progenitor cells, which were circulating in the blood stream and were directed to injured vasculature [10, 20]. Difficulties in correct identification of these ECs and controversial results on the long-term incorporation into the vasculature questioned the role of cEPCs as true physical building block contributing to new vessels and suggested that they mainly may act as paracrine source to promote vessel growth [144]. In a model of wire-injured carotid artery transplants, virtually all EC regeneration was mediated by migration of adjacent healthy ECs toward injury [32]. Today, the self-renewal and differentiation capacity of ECs upon injury are discussed to be mainly mediated by specialized ECs rather than invading cells. Whether increased endothelial plasticity can lead to shedding of ECs from damaged vasculature to the blood stream, or activation of migratory phenotypes (as it is known in metastasis), which may result in circulating populations of endothelial cells, is not fully clear. Taken together, the contribution of endothelial plasticity to healing processes, such as clonal expansion and migration, could largely be explained by overlapping similarities with cells of the hematopoietic system.

### Endothelial metabolic responses

The heart requires high amounts of energy and oxygen for ATP production. In fact, no other organ consumes metabolically so much energy as the heart. In homeostasis, the majority of ATP is generated via mitochondrial oxidative phosphorylation in cardiomyocytes. While the heart utilizes nearly all kind of energy sources, fatty acids are by far the most frequent [69]. Cardiac ECs have uniquely adapted to the organ demands as they express specific signatures of fatty acid transport, such as CD36 and fatty acid-binding proteins (FABP4, FABP5) [49, 95]. CD36 is required for endothelial up-take and transport of fatty acids to the neighboring cardiomyocytes. Partly, fatty acids are also retained and stored in ECs or used for own energy production. ECs, compared to other cells in the heart, have a relatively low number of mitochondria and do not require mitochondrial oxidative phosphorylation for ATP generation. This makes them more resistant to conditions of tissue ischemia and damage compared to cardiomyocytes [16]. Moreover, at high energy demands, ECs can utilize fatty acids, for DNA synthesis and cell division [62]. Under conditions of ischemia, the induction of HIF and oxygen-sensing mechanisms, such as eNOS or NADPH oxidases activity, augments glycolysis to maintain endothelial energy consumption in the low oxygen environment [7], while fatty acid metabolism is reduced [124]. This adaptation is mediated by various changes in the gene expression program leading to higher abundance of GLUT1 or PFKB3 [77] and repression of transcription factors such as FOXO1, which restrict glycolysis in quiescent ECs [136]. However, sufficient ATP generation in low oxygen levels is not the only advantage of a glycolytic program. Byproducts, such as glucose-6-phosphate, are fueled into the pentose-phosphate pathway to generate ribose-5-phosphate, a rate limiting source for nucleotides, which are required for proliferation. Additionally, hypoxia shifts glutamine metabolism from oxidation to reductive carboxylation [115]. In addition to the hypoxia and HIF-dependent changes in EC glycolytic and amino acid metabolism, genes encoding for fatty acid transport are among the strongest differential regulated genes in cardiac endothelial cells at the early stages after injury with the first 24 h infarction [124]. Loss of FABP4 and CD36 and induction of glycolysis-related genes is associated with increased plasticity and proliferation. In addition, loss of oxygen supply and high energetic requirements for angiogenesis promote de novo biogenesis and fusion of mitochondria [8, 91]. Increased demands for energy lead to ATP exhaustion, causing accumulating levels of AMP activates endothelial AMP-activated protein kinase (AMPK), which promote catabolic pathways in ECs in hypoxia after infarction [24, 137]. AMPK-mediated mitochondrial biogenesis, lipid metabolism and fat mobilization were shown to depend largely on SIRT1, another sensor of energy deprivation [75, 97]. Notably, in cardiac ECs, SIRT1 is known to be upregulated in ischemia induced neovascularization [100]. Downstream, SIRT1 represses FOXO1 and activates PGC-1A, which is known to induce mitochondrial biogenesis [14, 51]. D*e novo* formation of mitochondria was associated with an anti-inflammatory phenotype, and suppressed activity of TNF-α and NFκB [51]. However, it is unclear whether and how mitochondrial biogenesis causally contributes to transient EC adaptation and state changes.

### Endothelial-to-mesenchymal transition

Reactivation of developmental pathways can induce phenotypical changes and promote endothelial-to-mesenchymal transition (EndMT). This process has initially been described during valve formation, when ECs acquire mesenchymal characteristics by following TGF-β gradients in the cardiac jelly [82, 83]. Here, TGF-β induction leads to a complete and permanent lineage transition, as these cells continue to be fibroblasts. In adulthood, tissue damage following inflammation and hypoxia modulates the perivascular space favoring similar, yet rather incomplete processes. The degree of transition to mesenchymal lineage after myocardial infarction is highly debated. Initially, ECs undergoing EndMT were thought to contribute to myofibroblasts and hence to fibrosis [141]. Recently, however, single-cell sequencing revealed that mesenchymal gene identity at day 7 post infarction is present in ECs but remains rather insignificant [64]. Parallel lineage tracing of endothelial and mesenchymal identities revealed no EndMT after myocardial infarction depicted by α-SMA and ZEB1 expression [142]. Further studies using more detailed time course analysis raveled that ECs have distinct transcriptional response program to TGF-β in very early phases after injury. This transient mesenchymal activation (EndMA) was reversible at later stages when TGF-β level decline, indicative of a temporal and incomplete process [124]. In contrast to EndMT, cells undergoing EndMA showed only modest up-regulation of mesenchymal genes and no complete transition to fibroblasts or other mesenchymal cells [64, 124]

How is this phenotype initiated? Several cytokines in combination with hypoxia initiate EndMA. TGF-β, produced from cells of the innate immune system in response to tissue damage is one of the most potent inducers. Cardiac macrophages selectively express MMP14, which activates latent TGF-β and induce paracrine signaling in ECs post injury [5]. Additionally, many other drivers of EndMA have been identified, including turbulent flow, hypoxia, glycolysis, WNT signaling, NOTCH signaling or reduction in FGF signaling. Interestingly, pro-inflammatory NFκB activation in EC, as it occurs upon IL-1β stimulation augments transition into a mesenchymal phenotype [76, 143] and induces generation of TGF-β1 and TGF-β2 [86].

ECs undergoing mesenchymal activation after infarction were more glycolytic than naïve ECs, which maintain oxidative phosphorylation and fatty acid transport [124]. In fact, reduced fatty acid oxidation by decreased levels of CPT1A has been identified as inducer of EndMT transition [139]. Increased levels of cytoplasmatic acetyl-CoA suppressed induction of EndMT, indicating that plastic transition toward mesenchymal lineage is inhibited in ECs which maintain normal ATP levels and citric acid cycle activity. In addition, genes regulating fatty acid metabolism and mitochondrial biogenesis such as SIRT1 are counteracting TGF-β induction in ECs [68]. In contrast, increased levels of MYC, a main transcription factor which virtually drives expression of all glycolysis-related genes, promotes EndMT under hypoxic conditions [2].

Another mediator of EndMT is mitochondrial dysfunction and oxidative stress. Elevated levels of ROS promote not only the activation of NFκB, but also suppress KLF4 which deregulates endothelial identity and promotes EndMT [88, 107]. Conversely, hydrogen sulfide, a potent inhibitor of EndMT and scavenger of ROS species was shown to suppress TGF-β signaling [119]. Elevated levels of H_2_S are well known to induce an anti-inflammatory phenotype in ECs. This suggests that ECs, which are close to the injury site, are characterized by high level of ROS, glycolysis and mitochondrial dysfunction and share an inflammatory phenotype, which favors activation of TGF-β and hence EndMT. It seems reasonable that prolonged inflammation could lead to continuous transition to a complete mesenchymal phenotype and eventually fibrosis. Likewise, the reversible nature of EndMA after myocardial infarction might be a consequence of high endothelial resilience against inflammation and an active conversion to a naïve state. However, how ECs can switch back to the endothelial phenotype is poorly understood.

### Cardiomyocyte gene expression in endothelial cells

Single cell sequencing studies have consistently reported the expression of cardiomyocyte-specific gene expression signatures in ECs [64, 124]. While it is still debated whether these detected subpopulations are existing or may be the consequence of mRNA contamination during the single cells isolation procedure [1] some hints point to a possible activation of tissue-specific genes in ECs. Interestingly, Ribo-Seq of brain ECs revealed specific expression of synaptic genes, such as pleiotrophin [47]. In the heart, ECs show open chromatin of certain cardiomyocyte signature loci, express myofibrillar genes and cardiac-specific transcription factor MEF2C [70, 140]. Co-culture of human-induced pluripotent stem cell-derived cardiomyocytes with ECs induced MYL7 and MYL4 expression as well as NOTCH and BMP signaling in endothelial cells [35]. The functions of cardiomyocyte gene expression in healthy or injured endothelium however are unclear.

### Angiogenesis and proliferation

Neovascularization requires either sprouting angiogenesis of existing vessels or de novo formation of new blood vessels in the damaged area. Several cell types and origins have been discussed to infiltrate and expand in the tissue upon injury and contribute to new blood vessel formation.

#### Endocardial cells

Lineage tracing in early post-natal stages revealed significant contribution of endocardial cells to coronary endothelial cells [122]. Hence, endocardial cells were discussed to give rise to ECs after infarction too. Here, Cx-40-based lineage tracing revealed increased plasticity of endocardial cells post infarction concomitant with accumulation of arterial foci (endocardial flowers) which share angiogenic capacity [84]. Contrarily, studies using Npr3-Cre lines revealed that adult endocardial cells, unlike their neonatal counterparts, only minimally contribute to coronary EC populations after myocardial infarction [117]. The presence of trabecular myocardium, a feature of the developing heart which gets lost in adulthood might be required for complete endocardial to endothelial transitions [111]. Vice versa, paracrine activities of ECs are required for development of trabecular myocardium. If such paracrine signals are lacking this can result in left ventricular non-compaction pathologies [105, 131].

#### Fibroblasts as origin of new vessels

Fibroblasts have also been suggested to acquire endothelial cell phenotypes by undergoing mesenchymal-to-endothelial transition, thereby potentially contributing to de novo vascularization of the infarct zone [125]. However, lately the impact on neovascularization by increased plasticity of fibroblasts was questioned as other groups failed to detect endothelial cells of fibroblast origin in the heart, using PDGFRα or COL1A2 genetic tracing systems [34].

#### Progenitor cells

Single cell sequencing of cardiac endothelial cells described multiple novel subsets of cells, but tissue-resident cardiac microvascular progenitor populations have not been described [49, 64, 96, 124]. Moreover, the contribution of circulating blood-derived cells for generation of de novo formed vessels also has been challenged as discussed before “Endothelial responses to cell death, hypoxia and inflammation favor interactions with the immune system”. However, there is evidence of specialized EC populations, which reside alongside normal ECs and form new colonies upon tissue damage.

CD157 (BST1) was described to mark such a specialized “stem-cell” endothelial subset [132]. Notably, CD157^+^ CD200^+^ ECs were found in large vessels in multiple organs, but not in capillaries. Similarly, rare subpopulations of ECs in the damaged aorta show hyper-proliferative activity associated with increased levels of the bZIP activating transcription factors [83]. ATF3 and ATF4 demark angiogenic microvasculature in the skeletal muscle, most likely due to a metabolic priming, which is present in many subtypes of endothelial cells and not restricted to “stem cells” [22]. Whether these specialized cells contribute to neovascularization of the heart upon myocardial infarction remains to be determined.

#### Endothelial cells

Several studies suggest that cardiac microvascular ECs undergo plastic changes upon myocardial infarction and vascularize the infarct area. This includes degradation of the basal membrane, elongation toward a VEGF gradient and stabilization of the new vessel by pericytes. Based on the local environmental cues and the given vascular bed, two ways of sprouting angiogenesis appear to exist: random/stochastic contribution of ECs to the sprouts or the formation of truly new vessels by specialized hyper-proliferative cells which clonally expand. In contrast to post-natal retina angiogenesis, which was shown to occur in a stochastic manner, a clonal expansion of cardiac endothelial cells around the infarct area was noted [64, 78]. Interestingly, ECs from clonally expanded vessels showed a partial mesenchymal signature, suggesting that these cells have a privileged role within the EC population [78]. Interestingly, the proliferative potential is not uniform in EC subpopulations across the vascular bed. Postnatally, venous ECs reside in an early G1 phase, while arterial cells pause their cell cycle later at G1, which has implications for the proliferative potential and BMP and TGF-β signaling [17, 18, 81]. Interestingly, inducible deletion of the arterial genes ENG, ALK1 or SMAD4 leads to a hyper-proliferative phenotype associated with an up-regulation of venous specific genes [74, 92, 93]. These findings demonstrate that venous EC have a higher proliferation capacity and hint to a possible role of venous ECs as source for clonal expansion.

## ECs in fully regenerative models

To understand how efficient vessel growth can occur after injury and how EC resilience is controlled, we can take a look at fully regenerative species, such as zebrafish or at mice during post-natal stages. In zebrafish, re-establishment of the vascular network after cryoinjury occurs quickly but superficial, which may promote rapid oxygen and nutrient supply to the damaged tissue and additionally foster cardiomyocyte proliferation by 3D scaffold guidance [80]. Importantly, the regenerated vascular network was formed by pre-existing coronary vessels, which sprout toward the side of injury and inflammation. ECs around the infarct zone become glycolytic and activate proliferative programs concomitant with increased expression of Apelin, a known guidance molecule for sprouting endothelial cells [66, 80]. Collectively, sprouting guidance is augmented by hypoxia-dependent expression of *cxcl12* and *cxcr4* [79]. Increased EC paracrine signaling to the surrounding cells promote organ regeneration and drive cardiomyocyte proliferation to fully regenerate the infarcted tissue. Examples of such paracrine signals range from Wnt repression by Notch activation, Fgf signaling or activation of the endocardium via Pdgf. Moreover, epicardial-derived cells promotes the guidance of blood vessel formation by Cxcl12 and other mechanisms [71, 79]. Interestingly, similar increased EC paracrine signals can be also seen in mammalian responses to infarction [71].

While the regenerative capacity is tightly linked to reactivation of the cell cycle in cardiomyocytes, novel studies using single-cell sequencing revealed important differences in the injury response of endothelial cells to infarction in post-natal and adult mice. Compared to adult hearts, less lipocalin-2 (LCN2^+^) venous EC populations but higher expression of capillary EC markers were found, which could reflect the immature nature of neonatal blood vessels [134]. In addition, postnatally, epicardial-derived multipotent cells still reside in the epicardium and sub-epicardium [3]. It is clear, that ECs in fully regenerative models of myocardial infarction, such as zebrafish or neonatal mice, differ in the ability to induce a fast and efficient neovascularization of the damaged tissue. Specialized more immature cells, which might be absent in adults, may send paracrine signals to support angiogenesis and EC survival, which seems be key for initiate recovery.

## Mechanisms of enhanced endothelial resilience?

Translated on human behavior, the term *resilience* implies long-term successful adaptations to strong stressors or trauma [109]. This phenomenon represents not a personal trait, but rather can be seen as a summary of the individuals’ responses to biological or social factors which allows adaptive and self-protective behavior. The molecular resilience program of cells works indeed similarly. Resilience of ECs can be seen as the long-term successful adaptation to the combinatorial influence of different stressing factors after myocardial infarction. The onset of cell death, hypoxia, inflammation, changes in metabolism followed by plastic fate transitions and clonal expansion provides a selective and challenging pressure on the heart’s endothelium which might be beneficial for combating long-term heart failure.

Is there a way to increase resistance and resilience? Studies of the last decades have shown that ischemic pre-conditioning, an experimental technique with periodically repeated brief ischemia phases followed by extended reperfusion phases, prevent apoptosis and inflammatory responses [23, 98]. In humans, pre-conditioned individuals were less prone to endothelial dysfunction and neutrophil activation [50]. While many of the effects were eradicated after a limited timeframe post conditioning, there is evidence that some longer lasting structural changes in the vasculature take place.

As shown in different animal models, ischemia in the hind limb also led to beneficial and survival-prolonging effects in subsequent cardiac ischemia [135]. This remote pre-conditioning, utilizes humoral and neuronal mechanisms [38] to protect virtually all organs for secondary ischemic injury [13, 40, 42]. The underlying mechanisms are not fully understood but converge to the global induction of early response cascades of cell stress, such as ROS signaling or adenosine, bradycardic agents or cytokines [37, 58]. Downstream, pre-conditioning leads to altered JAK/STAT3 signaling in ECs which in turn has potential effects on survival pathways, apoptosis and proliferation [31, 60]. Ultimately, cells are likely to establish a mitochondrial protection phenotype, characterized by inhibition of the mitochondrial permeability transition pore (mPTP) [30, 36]. Mitochondria protection may be in part mediated by pre-conditioning induced elevation of mitochondrial telomerase reverse transcriptase, which improves EC functions [4].

Likewise, physical exercise has been described to directly modulate endothelial function by modulating NO bioavailability [133] and decreasing mitochondrial-derived oxidative stress [27, 52]. Training can significantly reduce consequences of aging by decreasing ROS production and increasing anti-oxidative protective mechanisms. This is accompanied by augmented levels of PGC-1α [99]. Curiously, moderate exercise levels influence the vascular protective role to oxidative stress by mildly augmenting ROS levels [89]. This triggers the induction of anti-oxidative signaling, e.g., by augmenting Nrf2 and counteracts existing cardiovascular dysfunction [93, 104]. In addition, pharmacological interventions can interfere with oxidative damage and induce mitochondria protection to mitigate the inflammatory response. Inhibition of angiotensin II pathways are known for their anti-inflammatory and vasculo-protective effects [21] and may contribute to EC resilience. In addition, the anti-diabetic drug metformin was shown to prevent the formation of oxidative mitochondrial DNA and reduced inflammation-related dysfunction of vessels and the lung in other disease models [41, 138].

It may be interesting to gain more insights in long-term cardioprotective and the consequences of pre-conditioning mechanisms or exercise on EC plasticity and clonal expansion.

## What if resilience of ECs fails?

Myocardial injury has a major impact on EC phenotypes resulting not only in metabolic adaptation but also in partial fate changes. While it is extensively studied how the injury response is regulated, little is known regarding the resolution of the adaptive responses. Certainly, this is of major importance, since a chronically inflamed endothelium would result in microcirculatory dysfunction, a key pathophysiological component of chronic heart failure. While low levels of ROS act as signaling molecules and may allow temporal adaptation, chronic elevation of ROS, together with reduced NO bioavailability is seen as an early pathological step toward long-term dysfunction [48]. Oxidative stress induced loss of mitochondrial DNA integrity impairs vasodilative capabilities [53]. Likewise, a continuous lack of endothelial fatty acid transporters, as it occurs transiently after infarction, would result in insufficient fatty acid availability resulting in profound changes to cardiomyocyte metabolism [46, 72, 73, 118]. Also, failed metabolic feedback may influence how the vascular tree is built and favors functional shunting [101, 102]. Finally, a chronic mesenchymal activation, due to prolonged or permanent mesenchymal induction may lead to complete transition and, hence fuel cardiac fibrosis and microvascular dysfunction.

How is the transition back to a “healthy” endothelium regulated? One possibility is that the transition is mediated by the normalization of stimuli, which induced the changes in endothelial identity. Thus, a reduction of inflammatory cytokines and the restoration of oxygen supply resulting in a normoxic environment may be sufficient to de-activate ECs allowing the return to the original maintenance program and identify. This assumption is supported by in vitro findings, showing that the sole withdrawal of TGF-β reverted EndMT [124]. The limitation of inflammatory responses in ECs might be mediated by a higher abundance of anti-inflammatory cytokines, which induce resolution of inflammation. In support of a role of anti-inflammatory cytokines previous studies showed that IL-10 treatment improved vascular remodeling [113].

However, the resolution of inflammation may not occur efficiently during aging or in the presence of risk factors. Here, transient inflammation might turn into chronic inflammation a condition referred to as “inflammaging”. Moreover, it is unclear whether epigenetic changes that may occur during inflammatory activation can be fully be restored. Certainly, future work is needed to understand the transient nature of endothelial plasticity.

## Abbreviations

AMPK: AMP-activated protein kinase
cEPCs: Circulatory endothelial progenitor cells
ECs: Endothelial cells
EndICLT: Endothelial-to-immune cell-like transition
EndMA: Endothelial-to-mesenchymal activation
EndMT: Endothelial-to-mesenchymal transition
I/R: Ischemia reperfusion
MI: Myocardial infarction
NO: Nitric oxide
mTPT: Mitochondrial permeability transition pore
ROS: Reactive oxygen species
